# Efficient Invariant Features for Sensor Variability Compensation in Speaker Recognition

**DOI:** 10.3390/s141019007

**Published:** 2014-10-13

**Authors:** Abdennour Alimohad, Ahmed Bouridane, Abderrezak Guessoum

**Affiliations:** 1 Research Laboratory in Electrical Engineering and Automatic LREA, University of MEDEA, Ain D'heb, Medea 26000, Algeria; 2 School of Computing, Engineering and Information Sciences, Northumbria University, Newcastle Upon Tyne NE2 1XE, UK; E-Mail: Ahmed.Bouridane@northumbria.ac.uk; 3 Department of Electronics Engineering, University of Blida, Blida BP 270, Algeria; E-Mail: abderguessoum@yahoo.com

**Keywords:** speaker recognition, invariant features, MFCCs, GMM-UBM, sensor variability, DET curve

## Abstract

In this paper, we investigate the use of invariant features for speaker recognition. Owing to their characteristics, these features are introduced to cope with the difficult and challenging problem of sensor variability and the source of performance degradation inherent in speaker recognition systems. Our experiments show: (1) the effectiveness of these features in match cases; (2) the benefit of combining these features with the mel frequency cepstral coefficients to exploit their discrimination power under uncontrolled conditions (mismatch cases). Consequently, the proposed invariant features result in a performance improvement as demonstrated by a reduction in the equal error rate and the minimum decision cost function compared to the GMM-UBM speaker recognition systems based on MFCC features.

## 1. Introduction

**Figure 1.**
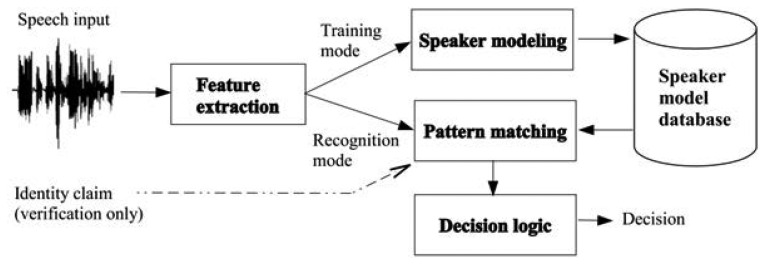
Speaker recognition system.

Biometrics refers to methods for uniquely recognizing humans based on intrinsic physical or behavioral characteristics. It offers a promising approach for security applications, with some advantages over the classical methods, which depend on something you have (a key or a card) or something you know (a password or a PIN). Speaker recognition is one of the emerging biometric technologies used for authenticating and monitoring human subjects using their speech signals [1]. It is attractive for two main reasons: (1) it does not require direct contact with the individual, thus avoiding the problem of “perceived invasiveness” inherent in many biometric systems, like iris and finger print recognition systems; (2) signal transducers (microphones) are now ubiquitous on most portable devices (cellular phones, PDAs and laptops). Speaker recognition covers identification and verification. In speaker identification, no identity is claimed from the speaker. The automatic system must determine who is speaking. If the speaker belongs to a predefined set of known speakers, it is referred to as closed-set speaker identification. The more general situation where the system has to deal with speakers that perhaps are not modeled inside the database is referred to as open-set speaker identification. In speaker verification, the goal of the system is to determine whether a person is who he/she claims to be. This implies that the user must provide an identity, and the system just accepts or rejects the users according to verification success or failure. Figure 1 illustrates a speaker recognition system where the only difference between identification and verification is how the decision module deals with inputs to produce the appropriate output [2].

Another type of classification divides the speaker recognition process into text-dependent or text-independent systems. The first class is a constrained mode, where the speech is either fixed (password) or prompted (please say “1 – 2 – 3”); this knowledge can improve the system performance. The text independent class is an unconstrained mode where the speaker must be recognized regardless of the text pronounced, which presents a more difficult challenge.

Recently, research in speaker recognition has been concentrated on addressing the variability problem. This variability is caused by channel, noise and temporary speaker characteristics (mood, tiredness, *etc.*). The term, session variability, refers to all of the phenomena that cause two recordings of a given speaker to sound different from each other. The mismatches in sensor, language, style of speech and environment between the training and testing affect dramatically the performance of the recognition system. Several methods have been developed to address this problem by acting on the feature extraction stage, model stage or decision stage. These methods include normalization techniques, feature mapping, speaker model synthesis, factor analysis and nuisance attribute projection (NAP) [3]. It is clear that an appropriate selection of features can significantly increase the classification performance. Invariant features, an alternative technique, have been successfully applied to a wide range of image analysis [4]. Invariant structures were proposed in speech recognition to overcome the non-linguistic factors [5,6]. Experimental results on connected Japanese vowel utterances show that these features achieve better recognition rates. Recently, Müller *et al.* [7] used invariant integration features (IIFs) in speech recognition and showed their good performance. In their work, they proposed features that are invariant to translation along the sub-band space (related to frequency), where the objective was to recognize the same speech, even when uttered by different speakers (speaker independent). In our work, we propose to use this idea to recognize the same speaker, even when the speech is recorded in different ways or in different environments.

## 2. Problem Formulation

In speaker recognition, the good choice of features that really represent the speaker is a very important step, especially in a situation of variability, where the data recording conditions are not the same between training and testing phases. The recognition performance degrades significantly

In our work, we propose to use the invariant features to recognize the same speaker, even when the speech is recorded in different ways or in different environments. In addition to the fact that the features are invariant to frequency translations, as in [7], they must be invariant to magnitude changes, too, and the paper considers the invariance of features to magnitude, as well as frequency variations.

**Figure 2.**
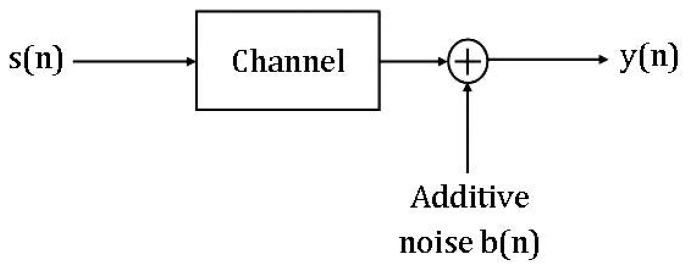
Process to obtain the observed speech.

The idea of these invariant features is to find a mapping T that is able to extract features that are similar for different observations x of the same equivalent class (speaker). Formally, we assume that clean speech is corrupted by a channel and additive noise (Figure 2). We have:

(1) y[n]=h[n]*s[n]+b[n]

where y[n] is the observed speech, h[n] is the impulse response of the channel, s[n] is the clean speech and b[n] is the additive noise. ⊛ denotes the convolution operation.

Note that the clean speech s[n] can be seen as a source signal, produced by the human glottal airflow, filtered with the resonances in the cavities of the human vocal tract. Taking the DFTand assuming that the frame size is of sufficient length compared to the length of the channel impulse response, we get:

(2) Y[k]=H[k]S[k]+B[k]

where *k* is the frequency index.

Equation (2) shows the effects of speaker characteristics (expressed by S(k)), the channel (expressed by H(k)) and the noise (expressed by B(k)) on the resulting speech signal Y(k). Each of those effects had been more or less intensively studied. Hansen *et al.* and Yao *et al.* [8,9], for example, studied the speech variability caused by stressful conditions. Ning *et al.* [10] designed a robust speaker recognition system to alleviate the noisy environment problem.

In this study, we consider the other important cause of variability due to the use of different channels, usually referred to as mismatch variability. We have developed a more robust speaker recognition system, by means of invariant features, which, to our knowledge, have not been used before in this area. However, to deal with the problem of magnitude changes, the proposed features are extracted according to both frequency translation and amplitude changes.

The following section describes in detail the invariant features technique. Section 4 gives a brief overview of a speaker recognition system by describing the feature extraction module based on mel frequency cepstral coefficients (MFCCs), the modeling module based on Gaussian mixture models and universal background models (GMM-UBM) and the decision module. Then, we describe the experiments and results obtained, and finally, a conclusion is given.

## 3. Proposed Invariant Features

In the following section, we introduce some basic terms and definitions from [11] that will be used later in the paper.

### 3.1. Definitions

The signal space S is a subset of a finite dimensional complex vector space V. We call the elements of S patterns and denote them by vectors, *i.e.*, v, w.

G is a group acting on V by a linear operator g. The action of G introduces an equivalent relation ∼ in S. Two patterns v, w are called equivalent, v ∼ w; then, gv = gw.

In our problem, the spectrally changed version of the same speaker should result in the same feature vectors.

We call the subset O(v) = {*g*v | g ∈ G } for a given v an orbit of G in S. The orbit contains all possible patters within one equivalent class. Two orbits *O*_1_ and *O*_2_ are either identical or have no point in common. A complete classification of that set of orbits lead to an exact discrimination between different classes; on the other hand, incomplete classification may lead to the same features from patterns of different classes. In practice, a “high degree of completeness” is desired. A possible solution for this problem can be addressed by the construction of a complete feature space F. This is a subset of some complex vector space together with a map T : S → F with the following properties:

(P1) T(v) = T(*g*v) for all g ∈ G, v ∈ S;
(P2) T(v) = T(w), then there exists a g ∈G with v=gw.

The condition P1 guarantees that equivalent patterns are mapped into one point, while P2 ensures that non-equivalent patterns are mapped onto distinct points in feature space.

### 3.2. Invariant Feature Spaces Construction for Speaker Recognition

#### 3.2.1. Background

Let T(*υ*) = (*f*_1_ (*υ*), …, *f_n_* (*υ*)), n ∈ N, be the components of the maps *f*, where *f_i_* : *S* → ℂ. These components *f_i_* are considered polynomials to be implemented on a computer.

Various methods for constructing such invariant features are known, e.g., normalization approaches, like moments [7], where the patterns are transformed with respect to an extreme point of the orbit, differential approaches where the invariant features are obtained by solving partial differential equations, such that the features remain invariant for infinitesimal variations of the parameter(s) of the group action or averaging methods [11].

In this final category of methods, the features are constant on orbits. Therefore, they describe properties that are common to all equivalent patterns. This suggests constructing features as appropriate averages. We try to calculate the averaged function *A_f_*(*υ*) by integrating f over the orbit *O*(*υ*):

(3) $$A_{f}(\text{v})=\int_{O(\upsilon )}f(g\text{v})dg$$

We call *A_f_* the G-average (or group average) of *f*.

This approach has been applied in speech and image processing [4,7]. This has motivated us to investigate it in the context of speaker recognition.

According to Noether's theorem [11], one can construct a basis of C-valued polynomials *f* defined on space S and which is invariant under the action of G on S (denoted by *f* ∈ ℂ[*S*]*^G^*) by calculating the group average of all monomials $\upsilon_{0}^{b_{0}}\upsilon_{1}^{b_{1}}\ldots \upsilon_{n-1}^{b_{n-1}}$ with *b*_0_ + *b*_1_ + … + *b_n_*_−1_ ≤ |*G*|, where |*G*| is the order of the finite group G.

Assume that the signal space S contains only a finite number of patterns. Let G be a finite group acting on S. Then, there exists an invariant *f* ∈ ℂ[*S*]*^G^* with the properties P1 and P2. The proof can be found in [11].

#### 3.2.2. The Proposed Feature Extraction Technique

In the case of speaker recognition, the signal is represented by a time frequency (TF) *υ_k_*(*n*), where 1 ≤ *n* ≤ *N* is the time index and 1 ≤ *k* ≤ *K* is the frequency index. The vector **v** = (*υ*_1_, *υ*_2_, …, *υ_K_*), which contains all spectral values for a specific time, known as frame in speech, is called the pattern.

As shown in Section 2, the variability may affect the component of vector **v** in amplitude and/or in frequency.

Consider, for example, two speech signals of the same speaker, one recorded by a certain type of microphone and the second recorded by another type of microphone. The first recording will be characterized by a channel transfer function *H*_1_[*k*], while the second recording will be characterized by a channel transfer function *H*_2_[*k*]. The resulting speech signal of each recording can be expressed in the frequency domain using the Equation (2) as follows:

$$\begin{array}{ll} Y_{1}[k] & =H_{1}[k]S[k]+B[k]\\ Y_{2}[k] & =H_{2}[k]S[k]+B[k] \end{array}$$

Using the vector representation introduced in this section, we associate a vector **v** to *Y*_1_ and a vector **w** to *Y*_2_. Since these two vectors represent the same speaker, an adequate choice of a group acting G yields to the property P2 of invariant features:

T(v)=T(w),then there exists a g∈G with v=gw

This action G depends on the nature of effects caused by the variability considered. In our work, the database used for speaker recognition allows us to study the mismatch variability, since for the same speaker, some recordings are taken by mobile phones and the others are taken by laptops (for more details, see Section 4.1).

**Figure 3.**
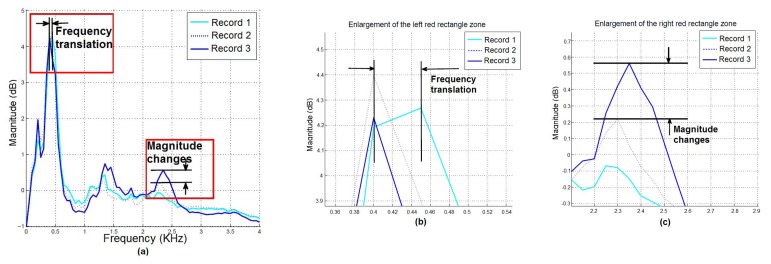
Spectral magnitude representation. (**a**) The entire spectra for the three records; (**b**) illustration of how frequency can change from one record to another for the same speaker; (**c**) illustration of how magnitude can change from one record to another for the same speaker.

To have a clear idea about the possible differences between many recordings of the same speaker, we represent in Figure 3 the spectra of one speaker recorded three times in different conditions.

We can see from Figure 3a that even if there are some similarities between these curves, differences in frequency, as well as in magnitude can be observed. The frequency change effect between Recording 1 and Recording 2 is clearly illustrated in Figure 3b, while the amplitude change effect between Recording 2 and Recording 3 is illustrated in Figure 3c. Therefore, these effects can be attributed to the action of the group G. They correspond to the translation operation for the frequency and the multiplication operation for the magnitude.

Formally, the spectra *S* of the same speaker in different conditions, A and B, are related by:

(4) $$S_{A}(\omega )=C(\omega ).S_{B}(\omega +\phi (\omega ))$$

where *φ* represents the frequency translation and C the magnitude change of the spectral component. Let *υ_k_*(*n*) denote the TF representation of a speech signal. As mentioned in [7], periodic boundary conditions have to be used, because they were required by the applied invariance transformations. In this paper, we use repeated boundary conditions:

*υ_k_*(*n*) = *υ*_1_(*n*) for *k* < 1, *υ_k_*(*n*) = *υ_K_*(*n*) for *k* > *K*, *Cυ_k_*(*n*) = 0 for *Cυ_k_*(*n*) < 0 and *Cυ_k_*(*n*) = 1 for all *Cυ_k_*(*n*) > 1, where 0 ≤ *υ_k_*(*n*) ≤ 1.

By assuming that a finite set of translations and multiplications along the spectral component space describes the effects of speaker variability, a finite group G of order |G| can be defined. Then, a group average is given by:

(5) $$A_{f}(\upsilon )=\frac{1}{\left|G\right|}\sum_{g\in G}f(g\text{v})$$

In our case of speaker recognition, *f* transforms the original spectrum *υ_k_* to *Cυ_k_*_+_*_i_*, as shown in Equation (4). The invariant features are defined as a group average on the basis of monomials m, such that:

(6) $$A(\upsilon )=\frac{1}{2W+1}\sum_{i=-W}^{+W}m(\upsilon ,i)$$

where *W* ∈ *N*_0_ is the window size. $m(\upsilon ,i)=\prod_{k=1}^{K}C\upsilon_{\left(k+i\right)}^{b_{k}}$is the set of the monomials m of v where *b_k_* ∈ *N*_0_, *i* ∈ *Z* and *C* ∈ ℝ. The order of a monomial is defined by the term $\sum_{k=1}^{K}b_{k}$. As an example, we consider the monomial m of order one, then, b*_k_*=0 for all k ∈ {1, 2, …,*K*}\*k*_1_ and *b_k_* = 1 for k = *k*_1_.

**Figure 4.**
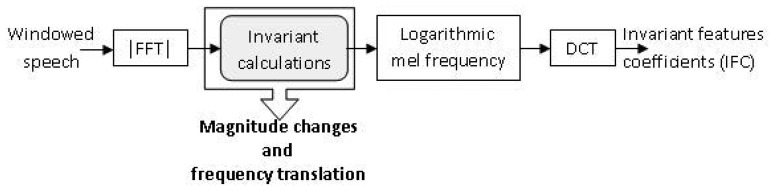
Steps for computing invariant feature coefficients.

Figure 4 summarizes the complete operation of invariants feature coefficient (IFC) extraction. As explained in this section, spectrum magnitude changes and the frequency translation actions are applied to obtain a set of invariant features. For each speech frame, we compute the magnitude FFT; we act on the resulting spectrum by changing the magnitude and frequency. Monomials of order one are then computed according to Equation (6), which is equivalent to computing the mean spectral value for a certain frequency k. It is clear that computing the mean spectral value does not change the spectral nature of the monomials. We then take the logarithmic magnitude of the mel frequency spectrum, and finally, a DCT transform is applied to obtain reduced and uncorrelated vectors suitable for the GMM-UBM technique. We call these IFC parameters.

## 4. Experiments

### 4.1. Database and Experimental Protocol Description

Our experiments were performed on the MOBIO database [12,13], which consists of speech data collected from 152 people (100 males, 52 females) using mobile phones and laptops. The mobile phone used to capture the database was a Nokia N93i, and the laptop computer was a standard 2008 MacBook. For each device, the data were recorded in a high quality format with a sampling frequency of 48 kHz. In addition to the noisy data, this database has many challenges, such as: session variability, lexical mismatch, speech-type mismatch, site mismatch and handset mismatch [12].

We followed the same protocol given in [12,13] by splitting the database into three distinct sets: training set, development set and testing set. The three sets are completely separate in terms of speakers and data collection sites (data are collected from six different universities). The background models were derived from the training set. The purpose of the development set was to derive the best parameters of the speaker recognition system, and the testing set was used to evaluate the system performance using these parameters. The protocol for enrolling and testing were the same for both sets. Only five set response questions from one session could be used to enroll a client. Testing was then conducted on all 15 free speech questions from the other five sessions, equaling 75 testing utterances per client. When producing imposter scores, all of the other clients were used as imposters. The performance was calculated in terms of equal error rate (EER) on the testing set and the minimum detection cost function (minDCF).

### 4.2. Description of the Speaker Recognition System

#### 4.2.1. Feature Extraction

The extraction and selection of the best parametric representation of acoustic signals is an important task in the design of any speaker recognition system; it significantly affects the recognition performance. Many types of features have been investigated [14]. Linear prediction coefficients (LPCs) have received special attention in this regard, as they are directly derived from the speaker's speech production model. Likewise, perceptual linear prediction (PLP) coefficients are based on human perceptual and auditory processing. However, over the past two decades, spectral-based features, derived by direct application of the Fourier transform, have become popular. These features are mel frequency-spaced cepstral coefficients (MFCCs), and their success arises from the use of perceptually-based mel-spaced filter bank processing of the Fourier transform and the particular robustness (to the environment) and flexibility that can be achieved using cepstral analysis.

**Figure 5.**
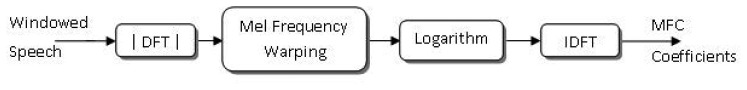
Steps of computing mel frequency cepstral coefficient (MFCC) features.

The key steps involved in computing MFCC features are shown in Figure 5. A discrete Fourier transform is applied to each windowed speech frame to yield complex spectral values. The global shape of the magnitude spectrum, known as the spectral envelope, which contains the information of the resonance properties of the vocal tract, has been found to be the most informative part of the spectrum in speaker recognition [15]. A simple model of spectral envelope uses a set of band-pass filters. The filters that are generally used in MFCC computation are triangular filters, and their center frequencies are chosen according to a logarithmic frequency scale, also known as the mel frequency scale. Furthermore, the sub-band energy values can be carried out in a perceptually meaningful way by smoothing logarithmically rather than linearly The final step is to convert these logarithmic spectral values into cepstral coefficients using the discrete cosine transform (DCT).

#### 4.2.2. GMM-UBM System

In the GMM-UBM system, we use a single, speaker-independent background model (also called world model) to represent *P*(*X/λ_UBM_*) [16]. The UBM is learned using data gathered from a large amount of speech. It is a large GMM, trained to represent the speaker-independent distribution of features. An iterative expectation-maximization (EM) algorithm is used to estimate the maximum likelihood model for the training feature vectors [17]. The estimates of the Gaussian mixture model parameters *λ_UBM_* = {*ω_UBM_, μ_UBM_, ∑_UBM_*} are improved at each iteration until convergence is reached.

We derive the speaker model by adapting the parameters of the UBM using the speaker's training speech and maximum *a posteriori* (MAP) adaptation [18].

### 4.3. Results and Discussion

All experiments use a gender-dependent GMM/UBM system; two UBM models, one for males and one for females, are trained using the total data of the two sessions (laptop session and mobile session) from two sites. The speaker models are obtained using the remaining sites, as explained earlier. This choice is justified by the fact that gender mismatches between training and test data are of no interest in our case, where we consider the sensor variability. We start by using only male models to study our proposed features in different situations. In the last experiment, we will show the performance results of speaker recognition for male and female trials. We use the ALIZE [19] open source toolkit to derive the UBM model, speaker models and log-likelihood ratios (scores). Five iterations of the EM (expectation-maximization) algorithm are used to model 512 mixtures of the UBM. The adaptation of the speaker's models is performed through one iteration of a MAP (maximum *a posteriori*) algorithm to adapt the mean values of each Gaussian. Our GMM/UBM baseline system uses 19 MFCC features. The system is based on 24 filter banks computed over 20-ms Hamming windowed frames on 8-KHz down-sampled signals (to match the phone case) with a 10-ms frame rate. The MFCCs are augmented with 19 delta, delta log-energy and 11 double delta coefficients. The total vector is so composed of 50 coefficients [20]. The proposed invariant features use monomials of the first order. The choice of this order has been explained in [7] and has proven to yield features that give the best performance. Similar to the MFCCs, we use 19 invariant features, augmented with 19 delta, delta log-energy and 11 double delta coefficients. In our work, the frequency translations is performed on all of the spectrum; while the multiplicative C acts on maximum amplitude by a change of 10%. We focus on the maximums, since the formants contain considerable information about the speaker's identity.

For performance evaluation, we plot the detection error tradeoff (DET) curve, known to be usefulness for many speaker and language recognition applications [21]. In the DET curve, we plot error rates (probability of miss *P_Miss_* and probability of false alarm *P_FA_*) on both axes, by giving uniform consideration to both types of errors. One of the most widely reported performance measures in speaker recognition is the point in the DET curve where *P_Miss_* = *P_FA_*; this point is called the equal error rate (EER). Another interesting point, called the minimum detection cost function [22], is also used. In all of our experiments, we represent each speaker by two models, one using a mobile phone and the other using a laptop. Then, we test the speakers for match and mismatch cases. In the first case, we will compare the speech recorded in a mobile phone with the model of a speaker recorded in a mobile phone, too. Then, in the second case, the experiment has been performed between speech recorded on a mobile phone and the model of a speaker recorded on a laptop.

**Figure 6.**
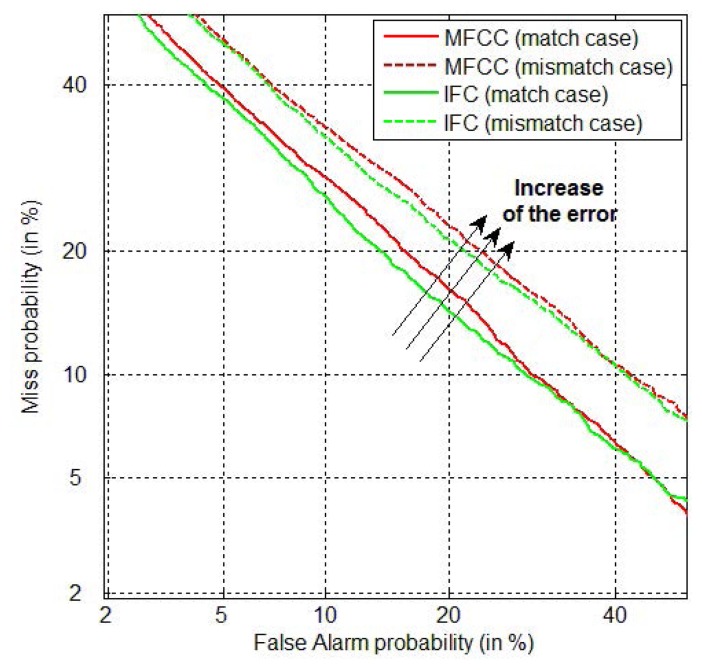
DET curves showing the performance degradation effect in the mismatch case (dashed graphs) compared to the match case (continued graphs).

The last two columns of Table 1 show the effect of using different kinds of handsets between testing and training phases, compared to using the the same kind of handset. It is clear that a net degradation of the system performance is observed in the mismatch case for all types of features (the MFCC and the proposed IFC). This effect of performance degradation is more detailed by the DET curve represented in Figure 6. We observe from this figure that the false alarm error rates and the false rejection error rates increase significantly in the mismatch case.

To illustrate the effect of using magnitude invariants in addition to the frequency translation invariants, we have compared the proposed features when using only frequency translations against a combination action of frequency and magnitude variations. The results depicted in Table 1 show the positive contribution of adding the magnitude, as shown by a reduction of the EER. This improvement is more obvious in different recording case between training and testing (mobile/laptop). Cepstral mean normalization (CMN) [15] is applied to the MFCC and the proposed invariant features. It is one of the simplest, but an effective way to remove time-invariant distortions introduced by the transmission channels. This was verified by the results shown in Table 1, where a significant improvement was achieved with CMN normalization. We have then applied this normalization to the rest of our experiments.

Compared to MFCC features, a performance improvement of the proposed features in both cases (the match case and mismatch case) was realized. This result confirms the effectiveness of invariant features in speaker recognition.

**Figure 7.**
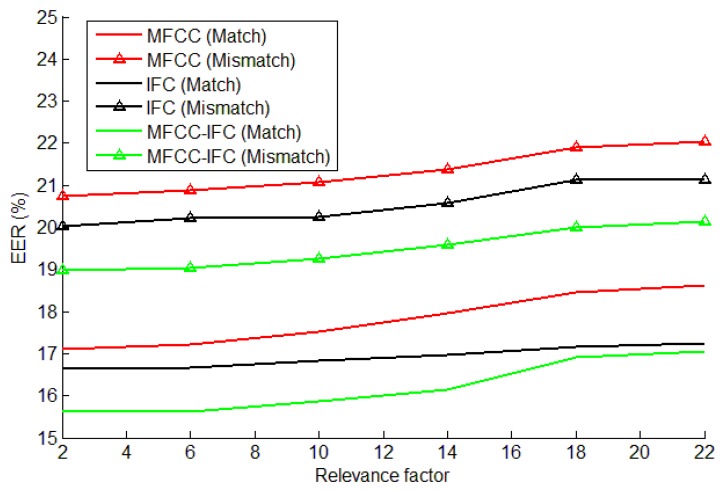
The effect of the relevance factor on system recognition performance in terms of EER.

The relevance factor controls how much new data should be observed in a mixture before the new parameters begin replacing the old parameters. In Figure 7, we show the effect of changing this factor on EER of the speaker recognition system. The best results were found when using values for the relevance factor between 2 and 10. We choose a value of six in all of our next experiments.

To take advantage of MFCC features in terms of their discrimination power and invariant features in terms of variability compensation, we propose to fuse the two methods together. A weighted linear combination of the MFCC scores and invariants scores are used as described in [23], and the new system scores are given by:

scores=β×scores(MFCC)+(1−β)×scores(IFC)

**Figure 8.**
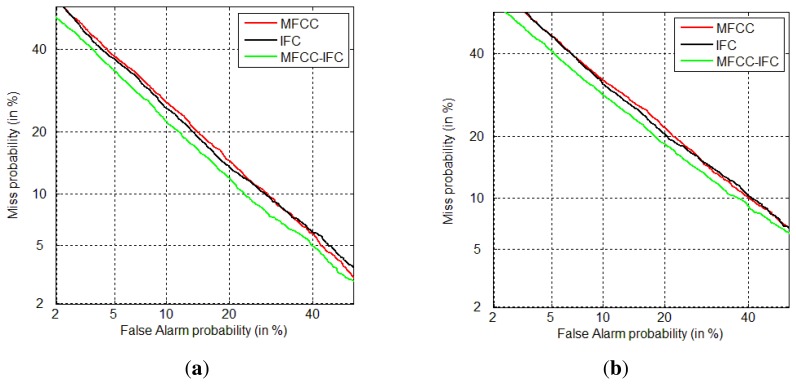
DET curves in the match case (**a**) and mismatch case (**b**).

In the context of speaker recognition based on the GMM-UBM system, the scores refer to the log likelihood function *P*(*X/λ*), where X represents the features vector extracted by the MFCC method for MFCC scores, while for IFC scores, X is extracted by the IFC method. Figure 8 shows DET curves of the three systems, based on MFCC, IFC and the fusion method. The left graph represents the match case, and the right graph represents the mismatch case.

We note that for the MFCC-IFC fusion method, a parameter *β* has to be adjusted. For this reason, we vary experimentally *β* between zero and one, until we get the best result (minimum error). The value corresponding to this result is obtained for *β* = 0.5. The curves corresponding to the MFCC-IFC method with this configuration are depicted in green color.

Table 2 gives the EER and minDCF for the proposed fusion technique based on the invariants and MFCC features.

As expected, an additional improvement is obtained by the fusion technique in both cases (the match and the mismatch cases). The degradation caused by using different recording conditions (mobile or laptop) is compensated with an EER improvement of almost 10% of the proposed fusion technique compared to the MFCC-based technique in the match and mismatch cases. In terms of minDCF, again, the proposed technique performs better with an improvement of more than 6%.

**Figure 9.**
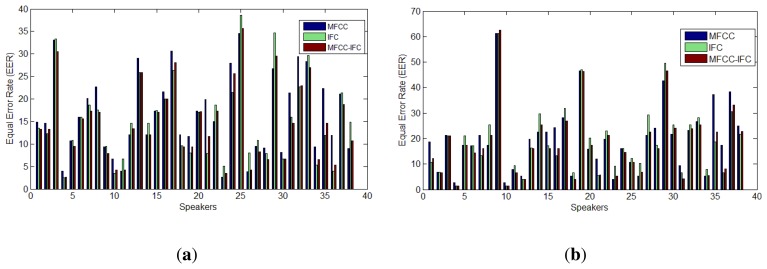
EER for each speaker (**a**) in the match case and (**b**) in the mismatch case.

To get better insight into the gain obtained by using the proposed technique in speaker recognition, Figure 9a,b represents the EER for each speaker of the tested database in the match case and the mismatch case, respectively. We calculate the EER and minDCF for each tested speaker, based on each speaker DET curve. Then, we take the mean of all of these resulting EER. From the figures, it can be observed that in the majority of cases, an improvement was achieved.

Table 3 gives the average EER and minDCF for the MFCC-based system, the proposed IFC and the proposed MFCC-IFC-based systems for male and female speakers. Once again, the results show the success of the proposed methods to reduce the EER and the minDCF compared to the MFCC method.

## 5. Conclusions

In this paper, we proposed the use of invariant features for speaker recognition by taking into account their successes in image and speech recognition. The goal of applying these features in the context of speaker recognition was to enhance system robustness against the variability problem. We started our work by studying the effect of mismatch between test and training on the speaker's spectrum. We then adapted the invariant features by adding the amplitude change invariance to the frequency translation invariance used earlier in the context of speech recognition. We applied these features in the GMM-UBM technique. Some improvements were obtained in both the controlled (match cases) and uncontrolled (mismatch cases) conditions when compared to the MFCC counterpart. Besides the variability compensation of the IFC features, to benefit from the discrimination power of the MFCC features, we combine the invariant features with the MFCC features. The results confirm our prediction, and an additional improvement is obtained.

## Figures and Tables

**Table 1.** Illustration of the speaker recognition system performance in term of equal error rate (EER) for MFCC and invariant features in match (same) and mismatch (different) cases with and without cepstral mean normalization (CMN) normalization. IFC, invariants feature coefficient.

	**Without**	**Normalization**	**With CMN**	**Normalization**
	
	**Same**	**Different**	**Same**	**Different**
EER (MFCC)(%)	19.21	25.75	17.96	21.39
EER (IFC)(%) (without magnitude action)	19.08	25.23	17.37	22.18
EER (IFC)(%) (with magnitude action)	18.36	25.06	16.94	20.59

**Table 2.** System performance of the fused MFCC-IFC method. minDCF, minimum detection cost function.

	**Same**	**Different**
EER (MFCC + IFC)	15.62	19.03
minDCF (MFCC + IFC) (×100)	6.79	7.41

**Table 3.** Mean EER and mean minDCF of individual EERs and the minDCFs of the speakers.

		**Female**		**Male**	
	
	**Same**	**Different**	**Same**	**Different**
EER (MFCC) (%)	17.40	22.11	16.05	18.94
minDCF (MFCC) (× 100)	6.77	8.08	6.71	7.26
EER (IFC) (%)	16.57	21.49	15.16	18.63
minDCF (IFC) (× 100)	6.65	7.97	6.55	7.21
EER (MFCC + IFC) (%)	16.36	20.85	14.19	17.33
minDCF (MFCC + IFC) (× 100)	6.40	7.84	6.29	6.80
